# Who is perceived as vulnerable? The role of gender conformity cues and explicit sexual orientation

**DOI:** 10.1111/bjso.70153

**Published:** 2026-11-10

**Authors:** Philipp Agostini, Morgana Lizzio‐Wilson, Mario Gollwitzer, Fabio Fasoli

**Affiliations:** ^1^ Department of Psychology Ludwig‐Maximilians‐Universität München Munich Germany; ^2^ Department of Psychology University of Exeter Exeter UK; ^3^ School of Psychology University of Surrey Guildford UK; ^4^ Centro de Investigação e Intervenção Social, ISCTE Instituto Universitário de Lisboa Lisbon Portugal

**Keywords:** gender conformity, intersectionality, male victims, sexual orientation, victim blaming, vulnerability

## Abstract

A substantial proportion of gay men report experiencing everyday harassment, raising questions about how observers interpret victimizing situations. Understanding these patterns requires examining how intersecting social categories shape perceptions of vulnerability, and how vulnerability is related to victim blaming. We investigated how minimal behavioural cues of gender conformity and explicitly disclosed sexual orientation jointly influence vulnerability‐related impressions. In Studies 1a/b (*N* = 248), gender non‐conforming targets were consistently perceived as more vulnerable and more susceptible to victimization. Studies 2a/b (*N* = 452) replicated these effects, while we found no significant effects of explicit sexual orientation on impressions of vulnerability and victimization susceptibility and no significant interactions with gender conformity. These findings highlight the consistent role of behavioural cues of gender conformity in shaping vulnerability‐related impressions when intersecting identity information is available. Moreover, perceived vulnerability was positively associated with both direct and indirect victim blaming, which suggests that vulnerability‐related impressions, rather than identity cues alone, are relevant to blame‐related judgements. Together, the results highlight how different sources of intersecting identity information are weighted when forming impressions of victimization and evaluating victims.

## INTRODUCTION

In Europe, 39% of gay men report having experienced everyday victimization because of their sexual orientation (45% in the United Kingdom; European Union Agency for Fundamental Rights, 2020). When gay men become victims, they often face disbelief, dismissal or blame by observers (Berrill & Herek, 1990) as they can be perceived as partially responsible for what happened to them (Kent, 2003). Gender non‐conformity is stereotypically associated with perceived gay sexual orientation (Kite & Deaux, 1987), and gender non‐conforming individuals are often stigmatized (Rodrigues, 2024). In addition, both gender non‐conformity and sexual‐minority status are associated with elevated victimization experiences (Mason et al., 2025; van Beusekom et al., 2020), with some evidence that their intersection is associated with greater victimization (Bränström et al., 2022; Martin‐Storey & Baams, 2019; van Beusekom et al., 2020). Importantly, sexual orientation is a unique social category as it can be both *inferred* from gender‐conformity cues (e.g. non‐verbal cues), which reflect gender prototypicality and gender expression rather than gender category alone, and explicitly *disclosed*. Together, these two sources of identity information provide an opportunity to examine how intersecting identities shape social perceptions.

Central to perceptions of victimization is vulnerability, which is linked to stereotypes (e.g. being fragile) signalling susceptibility to harm and the notion of the ‘ideal victim’ (see Walklate, 2011). Understanding how vulnerability and victimization susceptibility are attributed requires considering multiple identity cues and social category information in social perception. We examine whether gender conformity and sexual orientation influence perceptions of men's vulnerability and victimization susceptibility, and how these findings inform contemporary approaches to intersectionality by considering how non‐verbal cues and identity information are used to form impressions and judgements. We further explore how vulnerability ascriptions are related to victim blaming following harassment and how identity sources influence victim blaming.

### Gender conformity and sexual orientation

Gender is a perceptually ‘obvious’ social category that is easily accessible, with targets quickly categorized as men or women when perceptual cues (e.g. face) are available (Weißflog & Grigoryan, 2024; Zhang et al., 2018). Men are typically prototyped as masculine and women as feminine (Helgeson, 1994), and conformity to gender prototypes facilitates gender categorization (Fasoli et al., 2025; Weißflog & Grigoryan, 2024). Gender conformity is communicated through multiple cues, including gestures, gait and voice (Hardy et al., 2020; Ko et al., 2006; Lick et al., 2013) and represents a dimension of gender expression varying across gender and other intersecting categories (Sandfort et al., 2021).

Sexual orientation is an ‘ambiguous’ social category (Tskhay & Rule, 2013) as it cannot be known with certainty until the person self‐discloses. Nevertheless, individuals infer sexual orientation from perceptual cues (Rule, 2017). Because of its ambiguity, sexual orientation may not always be salient during person perception. Indeed, heterosexuality is often assumed by default (Lick & Johnson, 2016), establishing it as the norm and making it less salient unless there is a ‘deviation’. This ‘straight categorization bias’ has been observed when sexual orientation is inferred based on visual and vocal cues (e.g. Alt et al., 2020; Cox et al., 2016; Fasoli et al., 2023; Kachel et al., 2019; Rule et al., 2016). This bias occurs unless gendered non‐conformity is at play. In line with gender inversion theory (Kite & Deaux, 1987), gay men are stereotyped as less similar to the male prototype (i.e. less gender conforming), as they are associated with personality traits and interests that are stereotypically linked to women rather than men (Blashill & Powlishta, 2009; Klysing, 2023; Klysing et al., 2021; Mize & Manago, 2018). Such gender non‐conformity cues lead individuals to perceive a man as more likely to be gay than heterosexual (Freeman et al., 2010; Kachel et al., 2019; Rieger et al., 2010; Rule et al., 2016).

Thus, gender conformity can serve as a cue when *inferring* sexual orientation, whereas sexual orientation may also be explicitly *disclosed*. Hence, gender conformity and sexual orientation can intersect in ways that influence social perceptions. However, less is known about how impression formation operates when these are stereotype‐inconsistent (e.g. a gender‐conforming gay man or gender non‐conforming heterosexual man). Research on expectation violation provides some evidence. Gowen and Britt (2006) showed that a speaker who identified as heterosexual but sounded ‘gay’ was more stigmatized than a speaker who identified as gay and also sounded ‘gay’, suggesting that inconsistency between explicit and perceptual cues can influence evaluations. Still, their interplay has not been examined in relation to perceptions of vulnerability and victimization susceptibility.

### Vulnerability, victimization susceptibility and victim blaming

Vulnerability and victimization susceptibility can be inferred from perceptual characteristics. Non‐verbal cues, including gait, gestures and facial expressions, are not only seen as indicators of dominance and power (Hall et al., 2005; Richards et al., 1991; Rule et al., 2012) but also as signals of vulnerability (Book et al., 2013; Grayson & Stein, 1981). Targets who display vulnerability‐related cues are perceived as more likely to have been victimized in the past (Fulham et al., 2020; Wheeler et al., 2009). For instance, when a target's gait was manipulated to incorporate movements associated with perceived vulnerability (e.g. fluid lifting, shorter strides, less synchronous movements), participants attributed greater vulnerability and assumed that the target had a history of victimization (Blaskovits & Bennell, 2019; Gunns et al., 2002; Sakaguchi & Hasegawa, 2006). However, this research has largely overlooked perceptual cues signalling social category membership.

Social category information can matter too. Men are typically stereotyped as strong and capable of defending themselves (Van der Bruggen & Grubb, 2014), whereas gender non‐conforming men are perceived as weaker and less dominant (Martin & Slepian, 2020). Both gender non‐conformity and sexual minority status are associated with elevated victimization experiences (Mason et al., 2025; van Beusekom et al., 2020), with some evidence that the association between gender non‐conformity and victimization is particularly pronounced among sexual‐minority individuals (Bränström et al., 2022; Martin‐Storey & Baams, 2019; van Beusekom et al., 2020). However, this research has primarily examined social perceptions (e.g. dominance) or self‐reported victimization experiences (e.g. bullying), with little attention to how gender conformity and sexual orientation jointly shape social perceptions and evaluations when conveyed through perceptual or explicit cues.

Vulnerability and victimization attributions are closely tied to judgements of responsibility. Victim blaming is a pervasive social phenomenon across different forms of victimization, in which victims are held at least partially responsible for what happened to them (Kent, 2003). Contemporary blame theories propose that blame depends partly on whether individuals had the capacity and obligation to foresee and prevent harm (Malle et al., 2014; Niemi & Young, 2014). Moral judgements, such as victim blaming, are organized around perceptions of victimhood and harm, which serve as the primary template for evaluating whether an action is morally wrong (Gray & Kubin, 2024). Moral typecasting distinguishes between ‘moral agents’ and ‘moral patients’ (Gray et al., 2012; Gray & Wegner, 2009). Vulnerable individuals may be perceived as ‘moral patients’ – low in agency and high in susceptibility to harm – and as less responsible and blameworthy. Consequently, vulnerability should reduce victim blaming. However, vulnerability perceptions may also inform judgements central to contemporary blame theory, including expectations about individuals' capacity and obligation to foresee and prevent harm (Malle et al., 2014; Niemi & Young, 2014), potentially increasing blame when these expectations are violated.

Empirical research shows that victim blaming is influenced by a range of victim characteristics, including gender and sexual orientation. Male victims and gay men are often blamed more than their normative counterparts (e.g. Davies et al., 2006, 2008, 2009; Howard, 1984; Plumm et al., 2010), whereas evidence concerning gender conformity is limited. To our knowledge, only one study explicitly manipulated gender conformity, and did so by focusing on female victims and manipulating gender roles in written vignettes (Capezza & Arriaga, 2008), and one study considered sexual and gender identity by comparing straight, gay, cross‐dressing and trans targets without explicitly manipulating gender (non‐)conformity (Davies & Hudson, 2011). Moreover, this literature has focused predominantly on sexual violence, leaving open whether similar processes generalize to other forms of interpersonal victimization, such as verbal harassment. Together, these findings suggest that identity‐related cues, such as gender non‐conformity and gay sexual orientation, that increase perceived vulnerability and perceptions of victimization susceptibility, may contribute to stronger blame attributions, which is in line with the contemporary blame framework.

### Intersectionality in person perception

Research on intersectionality has examined whether intersecting social categories shape perceptions, stereotyping and evaluations. Our research focuses on men at the intersection of two main social categories: gender and sexual orientation. It also takes into consideration gender conformity, which reflects gender prototypicality and gender expression and serves as a cue for sexual orientation inferences (see gender inversion theory; Kite & Deaux, 1987; Miller, 2018).

Most research has examined intersections between social categories (e.g. gender and sexual orientation), with less attention to perceptual cues such as gender conformity that communicate category membership. Norm‐based models of intersectionality assume that certain groups (e.g. young adults, men, heterosexual people) represent the normative prototypes (e.g. Carnaghi et al., 2021; Lick & Johnson, 2016; Smith & Zárate, 1992). Hence, individuals with intersecting social categories (e.g. gay men) may be seen as deviating from these prototypes and as less representative of the broader categories. Indeed, Klysing et al. (2021) showed that gay men were seen as less prototypical than their main gender category (men; see also Klysing, 2023). Gender conformity is one cue through which gender prototypicality is communicated. Thus, gender non‐conforming gay men may be perceived as less prototypical of their gender category (men) because of their non‐verbal behaviour and sexual orientation.

In intersectionality approaches, person perception mechanisms (e.g. social categorization, stereotyping, salience) are key in understanding bias and social consequences (see Sternberg & Badea, 2026). Contemporary approaches do not assume that all intersecting identities contribute equally to perception but rather suggest that individuals are perceived and evaluated differently depending on which category becomes cognitively prioritized in a given context (see Hudson et al., 2024; Nicolas et al., 2017). To explain inconsistent findings, contemporary intersectionality models increasingly conceptualize person perception as a dynamic process in which the influence of different identities and identity cues varies across contexts. They recognize that identity cues may be more or less influential on person impressions (for stereotype dilution or amplification, see MOSAIC model, Hall et al., 2018) depending on their salience in a given context. This perspective is particularly relevant for sexual orientation, which can be inferred from perceptual cues of gender conformity or explicitly communicated via disclosure, making such an identity more or less explicitly salient.

As highlighted by Sternberg and Badea (2026), different models explain when and why intersectionality leads to different person perception and social consequences. Indeed, the dynamic interactive model (Freeman & Ambady, 2011) proposes that social perceptions are influenced by the dynamic integration of bottom‐up perceptual cues, competing social categories and stereotype activation, with different representations competing until a coherent impression is formed. This suggests that perceptual cues of gender conformity and explicit information about sexual orientation are integrated in social perceptions, although their influence depends on the context. Therefore, while an intersecting identity may not be suppressed, one category simply becomes more relevant. Building on these approaches, the intersectionality lens model (Petsko et al., 2022; Petsko & Bodenhausen, 2019) suggests that individuals adopt different interpretative lenses when perceiving and evaluating individuals with intersecting identities. Lens activation depends on *accessibility*, namely the ease with which schemas are activated, and *fit*, namely the relevance of the lens to the context. Other factors include perceived *goals*, which may motivate the use of a single or intersectional lens, and *distinctiveness*, which refers to the fact that a specific lens becomes very accessible because it is rare or unexpected. According to this model, stereotype activation depends on whether a single‐category or an intersection becomes cognitively prioritized in a given context (see Preddie & Biernat, 2021).

Our research informs intersectionality approaches by examining how behavioural cues signalling gender conformity and explicit sexual orientation information jointly shape perceptions and judgements related to victimization. By focusing on vulnerability, victimization susceptibility, and victim blaming, we examine whether perceivers prioritize different sources of identity information depending on their relevance to the context, as suggested by contemporary models of intersectional person perception (see Sternberg & Badea, 2026).

### The present research

To examine these processes, we conducted four studies. One aim of the research was to examine whether gender conformity cues elicit stereotype‐related perceptions of vulnerability in the form of attributed traits to the target, as well as event‐based inferences related to victimization susceptibility (i.e. attributed history, likelihood and ease of victimization) and whether these were shaped by *inferred* or *explicit* sexual orientation. Examining these outcomes separately is informative because vulnerability and victimization susceptibility may not be equivalent (e.g. Jackson, 2009). Our second aim was to examine whether intersectionality played a role in victim‐blaming judgements when the target man was described as a victim of verbal harassment.

Studies 1a and 1b focused on testing our first hypothesis that gender non‐conforming male targets would be perceived as more vulnerable (*H1.1*), more likely to have a history of victimization (*H1.2*), more likely to be victimized (*H1.3*) and easier targets of victimization (*H1.4*) than gender‐conforming male targets. In these studies, we also tested whether alignment to the male prototype signalled by gender conformity also prompted cross‐category inferences, namely assumptions about sexual orientation. By using gender conformity cues to infer sexual orientation, people rely on gender inversion heuristics to make assumptions about who is gay or straight (e.g. Freeman et al., 2010; Kachel et al., 2019; Kite & Deaux, 1987; Masi & Fasoli, 2022; Rieger et al., 2010; Rule & Alaei, 2016). Hence, we explored (RQ1) whether gender non‐conformity elicited perceptions of the target as gay, thus activating the intersecting category of ‘gay man’, and whether such perceptions mediated the relationship between gender conformity and perceived vulnerability.

However, sexual orientation can also be explicitly disclosed, making the intersecting social category salient. In Study 2a and 2b, we manipulated both gender conformity via minimal cues and sexual orientation by explicit disclosure, making the target's intersectional identity explicit rather than inferred. We assessed perceived vulnerability and victimization susceptibility, but also tested victim blaming after informing participants that the target had been a victim of harassment. Here, we tested Hypothesis 1 alongside additional hypotheses. We hypothesized that the effect of gender conformity would be moderated by explicit sexual orientation information (*H2*). More precisely, we expected that describing the target as gay (vs heterosexual vs. unspecified) would amplify the effect of gender conformity on perceived vulnerability (*H2.1*), attributed victimization history (*H2.2*), perceived likelihood of victimization (*H2.3*) and the attribution of being an easy target of victimization (*H2.4*). Moreover, we introduced the target as a victim of harassment and examined victim‐blaming. We explored the relationship between vulnerability perceptions and victim blaming (RQ2). Next, we predicted that gender non‐conforming male victims would be blamed more than gender‐conforming male victims (*H3*) and explored whether explicit sexual orientation moderated these blame judgements (RQ3).^1^

This research approach allowed us to understand whether perceivers prioritize single‐category or intersecting category information when forming impressions and making judgements related to victimization in line with recent models of intersectionality. Moreover, it considered situations in which social category is inferred or made explicitly salient and their influence on social perceptions.

The preregistration and analysis plan is available under https://osf.io/buzvq for Studies 1a and 1b and under https://osf.io/qzu6g/ for Studies 2a and 2b. All measures, analysis scripts, code and outputs, as well as Supplementary Materials, are available online at https://osf.io/c4qfm for all studies.

The studies were approved by the University Ethics Committee (UEC) of the University of Surrey (EGA ref.: 0116). Informed consent was obtained from all participants.

## STUDY 1

Study 1 tested whether gender conformity influences perceptions of vulnerability and victimization susceptibility. Across two versions of the study, we manipulated gender conformity using different stimuli. Research on minimal cue perception suggests that individuals are more confident and accurate in their judgements when a full video, rather than a single cue, is available (e.g. Ambady et al., 1999; Rieger et al., 2010). Moreover, gender conformity conveyed through gestures is particularly informative when multiple cues are available (Fasoli et al., 2026) and voice alone can trigger perceptions of gender conformity (Masi & Fasoli, 2022). Hence, we decided to test the same processes when gender conformity was conveyed either through multiple cues (Study 1a) or voice alone (Study 1b). Moreover, in line with gender inversion assumptions, gender conformity triggers sexual orientation categorization (Kachel et al., 2019), potentially making such social categories salient. Thus, we explored whether inferences of intersectionality were related to perceptions of vulnerability and victimization susceptibility.

### Study 1a

#### Method

##### Sample

An a priori power analysis (G*Power, Faul et al., 2007) indicated a sample of *N* = 102 was necessary to detect an effect of Cohen's *d* = 0.5 with *α* = .05 (one‐tailed, referring to the mean difference between the two conditions hypothesized in H1.1) and a power of 1 − *β* = .80. Participants were recruited through the SONA research participation system. After excluding 5 participants who did not complete the survey and 17 participants who failed the attention check, the final sample consisted of 104 (*M*_age_ = 19.34 years, *SD*_age_ = 1.62 years; 89 women, 14 men, 1 non‐binary; 76 heterosexual, 5 gay/lesbian, 5 bisexual, 3 ‘other’; 15.4% with university degree; 95.2% were students).^2^

##### Procedure and materials

After providing informed consent, participants watched a video clip used to manipulate gender conformity. An attention check assessed whether participants watched the video attentively (yes/no). Next, they rated the target in the video on perceived vulnerability and victimization susceptibility (scales presented in a randomized order). After rewatching the video, participants formed an impression about the man's gender conformity (manipulation check) and sexual orientation. Before being debriefed, participants reported their demographics.

##### Videos

Participants watched a video clip depicting young (between 25 and 35 years old) Australian Caucasian male actors wearing black T‐shirts while talking about neutral topics (i.e. weather and coffee). Two versions of each clip (12–15 s) were created, shortening the original videos by Gerrard et al. (2023), to portray gender conformity (GC) and non‐conformity (GNC) through stereotypically masculine or feminine non‐verbal cues (e.g. voice, posture and gestures).

##### Perceptions of vulnerability

We used a 6‐item scale (*α* = .78; Blaskovits & Bennell, 2019; Rule et al., 2012), asking participants to attribute traits (e.g. ‘vulnerable’ and ‘exploitable’) to the target on a scale from 1 (*not at all*) to 6 (*completely*). Items were averaged; higher scores indicated greater perceived vulnerability.

##### Victimization susceptibility

Three single‐item measures were included. We assessed perceived *history of victimization* (i.e. ‘How likely do you think this person has a history of victimization?’; 1 = *very unlikely*, 6 = *very likely*; self‐generated), *victimization ease* (i.e. ‘How easy/difficult do you think it is for this person to be victimized?’; 1 = *very difficult*, 6 = *very easy*; Gunns et al., 2002) and *victimization likelihood* (i.e. ‘How likely do you think this person is being victimized?’, 1 = *very unlikely*, 6 = *very likely*; self‐generated). An additional measure of victimization likelihood across different incidents is reported in Supplementary Materials.

##### Gender conformity (manipulation check)

Participants rated the target's masculinity/femininity on a 7‐point scale ranging from 1 (*very feminine*) to 7 (*very masculine*; Kachel et al., 2016).

##### Perceived sexual orientation

Participants rated the target's sexual orientation on a Kinsey‐type scale ranging from 1 (*exclusively heterosexual*) to 7 (*exclusively gay*).

#### Results

##### Gender conformity (manipulation check) and perceived sexual orientation

GNC men were perceived as less masculine and more likely to be perceived as gay than GC men (see Tables 1 and 2).

**TABLE 1:** Mean and standard deviations of all measured variables for all studies.

Variable	Study 1a		Study 1b		Study 2a		Study 2b	
	GC	GNC	GC	GNC	GC	GNC	GC	GNC
Perceived gender conformity	5.06 (1.21)	2.77 (0.87)	5.42 (1.05)	3.48 (1.11)	5.06 (1.21)	3.18 (1.25)	5.33 (1.13)	3.61 (1.45)
Perceived sexual orientation	2.76 (1.35)	5.55 (1.17)	2.15 (1.08)	4.76 (1.79)	2.94 (1.71)	5.12 (1.51)	2.38 (1.48)	4.59 (1.84)
Perceived vulnerability	3.07 (0.69)	3.50 (0.54)	3.05 (0.79)	3.56 (0.89)	2.94 (0.59)	3.54 (0.73)	3.04 (0.72)	3.54 (0.80)
Victimization history	2.96 (0.96)	3.81 (0.94)	2.68 (0.93)	3.45 (1.22)	3.02 (1.07)	3.80 (1.13)	2.79 (1.07)	3.65 (1.14)
Victimization ease	3.10 (0.90)	3.58 (0.82)	2.92 (1.02)	3.54 (1.13)	2.88 (0.95)	3.56 (1.03)	2.82 (1.08)	3.36 (1.22)
Victimization likelihood	2.98 (1.05)	3.75 (1.05)	2.60 (0.92)	3.32 (1.27)	2.72 (1.00)	3.45 (1.13)	2.70 (1.08)	3.35 (1.30)

*Note*: Perceived gender conformity and perceived sexual orientation were measured on 7‐point scales (1–7); all other variables were measured on 6‐point scales (1–6). Values represent mean values, with standard deviations in parentheses.

**TABLE 2:** Independent‐samples Welch *t*‐tests (one‐tailed: GC<GNC) for each DV.

Dependent variable	Study 1a			Study 1b		
	*df*	*t*	*d*	*df*	*t*	*d*
Perceived gender conformity	90.66	11.05	2.18*** [1.69, 2.66]	141.15	10.80	1.81*** [1.41, 2.19]
Perceived sexual orientation	98.76	−11.21	−2.21*** [−2.69, −1.71]	114.03	−10.56	−1.77*** [−2.16, −1.38]
Perceived vulnerability	94.78	−3.55	−0.70*** [−1.09, −0.30]	139.28	−3.66	−0.61*** [−0.94, −0.28]
Victimization history	101.68	−4.56	−0.90*** [−1.30, −0.49]	130.77	−4.24	−0.71*** [−1.05, −0.37]
Victimization ease	100.23	−2.88	−0.57** [−0.96, −0.17]	139.73	−4.43	−0.57*** [−0.90, −0.24]
Victimization likelihood	101.89	−3.75	−0.74*** [−1.13, −0.34]	127.53	−3.88	−0.65*** [−0.98, −0.31]

*Note*: Negative *t*‐ and *d*‐values indicate larger effects in the GNC condition (GC < GNC). Square brackets are used to enclose the lower and upper limits of confidence intervals.

** *p* < .01;

*** *p* < .001.

##### Main analyses

We tested our hypotheses using independent‐samples, one‐tailed *t*‐tests for each dependent variable. As shown in Tables 1 and 2, GNC men were perceived as more vulnerable and susceptible to victimization (i.e. higher victimization history, victimization ease and victimization likelihood) than GC men.

##### Mediation analysis

Although perceived sexual orientation was assessed after vulnerability‐related impressions, we explored its role as a mediator based on theoretically informed assumptions about the conceptual ordering of these variables. Specifically, we explored whether perceived sexual orientation mediated the effect of our gender (non‐)conformity manipulation on perceived vulnerability. Gender non‐conformity predicted perceived sexual orientation, *β* = .74, 95% CI [.61, .87], *p* < .001, which was significantly related to perceived vulnerability, *β* = .55, 95% CI [.30, .81], *p* < .001. Moreover, gender conformity was indirectly related to perceived vulnerability via perceived sexual orientation, *β* = .42, 95% bootstrap CI [.19, .70] (see Supplementary Materials for analyses on victimization susceptibility). Given the measurement ordering, this exploratory indirect effect should not be interpreted causally.

## Study 1b

### Method

#### Sample

Participants recruited through Clickworker, a crowdsourcing platform, took part in the study. After excluding 19 participants who did not complete the survey and 28 who failed the attention check, the final sample consisted of 144 participants (*M*_*age*_ = 41.31, *SD* = 10.26; 62 women, 79 men, 2 non‐binary and 1 ‘other’; 126 heterosexuals, 2 gay/lesbian, 12 bisexual and 4 ‘other’; 2.8% students; 70.2% with a university degree). The final sample fell below the preregistered target of *N* = 156 required to detect an effect of *d* = 0.4 (one‐tailed, mean difference hypothesized in H1.1) with *α* = .05 and 1 − *β* = .80.^3^

#### Procedure and materials

The procedure was identical to Study 1a, except that participants listened to an audio recording of a man instead of watching a video clip. All measures were the same as in Study 1a (perceived vulnerability scale: *α* = .86).

##### Audio recordings

Participants were presented with one audio recording of a man introducing himself (e.g. ‘Hi, I am Simon, I am interested in this job.’, 8 s). Participants were told that the audio was extracted from a job interview. Speakers were young (between 22 and 31 years old) British men from the Surrey region. We selected two GC and two GNC speakers from a larger pool (see Supplementary Materials for additional information).

#### Results

##### Gender conformity (manipulation check) and perceived sexual orientation

GNC speakers were rated as less gender‐conforming and more likely to be gay than GC speakers (see Tables 1 and 2).

##### Main analyses

GNC male speakers were perceived as more vulnerable, more likely to have a history of victimization, easier targets of victimization and more likely to be victimized in the future compared to GC speakers (see Tables 1 and 2).

##### Mediation analysis

We conducted the same mediation analysis as in Study 1a. Gender conformity predicted perceived sexual orientation, *β* = .66, 95% CI [.54, .79], *p* < .001, which was positively associated with vulnerability, *β* = .32, 95% CI [.11, .52], *p* = .003. The manipulated gender (non‐)conformity was indirectly associated with vulnerability via sexual orientation, *β* = .21, 95% bootstrap CI [.06, .38], which mirrors the findings from Study 1a.

### Discussion

Study 1 shows that minimal cues of gender (non‐)conformity are sufficient to shape vulnerability‐related perceptions. In line with Hypothesis 1, GNC men were perceived as more vulnerable, more likely to have a history of victimization, more likely to be victimized in general and as easier targets of victimization than GC men. Moreover, gender conformity cues made intersectional category membership salient, as gender non‐conformity elicited perceptions of the male targets as gay, and perceptions of sexual orientation were related to vulnerability perceptions. The same effects emerged both when voice was the only cue of gender conformity (Study 1b) and when multiple non‐verbal cues were available (Study 1a), suggesting that even a single cue serving as a proxy for gender prototypicality is enough for observers to infer intersectional identities and activate vulnerability‐related stereotypes.

## STUDY 2

Study 2 examined whether orthogonally manipulating gender conformity and sexual orientation influenced perceptions of vulnerability and victimization susceptibility beyond inferred sexual orientation, and which information was prioritized. While gender non‐conformity cues elicit inferences of gay sexual orientation, explicitly identifying GNC and GC targets as gay or straight creates multiple intersections that may be either stereotypically consistent or inconsistent, potentially affecting perceptions and judgements. Moreover, we introduced the target as a victim of harassment and assessed victim‐blaming judgements. This allowed us to investigate the association between perceived vulnerability and victim‐blaming.

### Study 2a

#### Method

##### Sample

An a‐priori power analysis (G*Power, Faul et al., 2007) indicated that a sample of *N* = 187 is required to detect a GC (2) × SO (3) interaction effect of $\eta_{p}^{2}$ = .05 with *α* = .05 (two‐tailed), and a power of 1 − *β* = .80. The sample was recruited through social media, SONA and Clickworker. After excluding 54 participants who did not complete it and 26 participants who failed the attention check, the final sample consisted of 233 participants (*M*_age_ = 25.15 years, *SD*_age_ = 9.82 years; 151 women, 80 men, 2 non‐binary; 208 heterosexual, 3 gay/lesbian, 19 bisexual, 3 ‘other’; 37.77% with university degree; 63.5% were students).

##### Procedure and materials

Before being exposed to the manipulations, participants completed measures of individual‐level variables (i.e. social dominance orientation, right‐wing authoritarianism, general belief in a just world), which were administered for exploratory purposes (see Supplementary Materials). Participants were then instructed to watch a video clip introduced as an extract from an interview with a male person (Andrew S.) who was described as heterosexual, gay or whose sexual orientation was not mentioned (*sexual orientation manipulation*). Next, participants watched the same video of Study 1a, manipulating GC or GNC (*gender conformity manipulation*). We assessed perceived vulnerability (*α* = .76) and victimization susceptibility (i.e. history of victimization, victimization ease and victimization likelihood), along with perceived gender conformity and sexual orientation as manipulation checks. All these variables were the same as in Study 1.

Participants were then asked to read an appeal for witnesses describing a verbal harassment incident in a local bar, in which Andrew S. (i.e. the man in the video) was approached by an unknown man and confronted with unsolicited comments (e.g. ‘I bet people get the wrong idea about you.’). In a pilot study, the scenario was perceived as moderately severe and relatively frequent (see Supplementary Materials).

Participants completed a measure of direct victim blaming (DVB) consisting of four items (e.g. This person got into this situation partly through his own fault.’; Agostini & Gollwitzer, 2026; *α*_DVB_ = .90). We also assessed indirect victim blaming (IVB), operationalized as a metacognitive task that would minimize social desirability, using parallel items phrased in third person (e.g. ‘Other people would agree that the person got into this situation partly through his own fault.’; *α*_IVB_ = .90). Answers were provided on 6‐point scales (1 = *completely disagree*; 6 = *completely agree*) and averaged to form an index, with higher values indicating stronger victim blaming. Participants then completed exploratory dependent variables related to the victimization incident (i.e. victim empathy, perceived severity, acknowledged suffering, moral outrage, perpetrator blame and responsibility; see Supplementary Materials). Finally, participants provided their demographics and were debriefed.

##### Results

###### Manipulation checks: Gender conformity and perceived sexual orientation

A 2 (gender conformity) × 3 (sexual orientation) between‐subjects ANOVA was performed on both variables.

For gender conformity, a significant main effect of gender conformity indicated that GNC speakers were perceived as less masculine than GC speakers. The main effect of sexual orientation and the interaction effect were not significant (see Tables 1 and 3).

**TABLE 3:** ANOVA results for relevant measured variables for Studies 2a and 2b.

Variable	Factor	Study 2a			Study 2b		
		*F*	*p*	$\eta_{g}^{2}$	*F*	*p*	$\eta_{g}^{2}$
Perceived gender conformity	GC	134.05	<.001	.37	94.81	<.001	.31
	SO	0.54	.584	.00	2.21	.112	.02
	GC × SO	0.10	.903	.00	0.24	.783	.00
Perceived sexual orientation	GC	113.97	<.001	.33	103.82	<.001	.33
	SO	7.08	.001	.06	6.95	.001	.06
	GC × SO	5.45	.005	.05	1.43	.242	.01
Perceived vulnerability	GC	50.28	<.001	.18	23.05	<.001	.10
	SO	2.79	.063	.02	1.91	.151	.02
	GC × SO	0.56	.570	.00	2.49	.086	.02
Victimization history	GC	29.83	<.001	.12	24.28	<.001	.14
	SO	2.74	.067	.02	1.38	.253	.01
	GC × SO	0.74	.480	.01	0.30	.739	.00
Victimization ease	GC	29.40	<.001	.11	14.18	<.001	.06
	SO	2.67	.071	.02	2.99	.053	.03
	GC × SO	1.86	.158	.02	0.91	.406	.01
Victimization likelihood	GC	28.39	<.001	.11	17.42	<.001	.08
	SO	2.76	.065	.02	1.83	.163	.02
	GC × SO	0.19	.826	.00	0.29	.749	.00
Direct victim blaming (DVB)	GC	0.00	.998	.00	2.52	.114	.01
	SO	0.38	.686	.00	0.36	.696	.00
	GC × SO	1.16	.316	.01	0.49	.611	.00
Indirect victim blaming (IVB)	GC	0.92	.340	.00	5.60	.019	.03
	SO	0.36	.697	.00	0.17	.848	.00
	GC × SO	0.33	.716	.00	0.85	.428	.01

*Note*: GC = gender conformity (conforming vs. non‐conforming); SO = sexual orientation (heterosexual vs. gay vs. unspecified). $\eta_{g}^{2}$ denotes generalized eta squared. Degrees of freedom are identical across analyses within each study (*df* = 1, 227 for GC; *df* = 2, 227 for SO and GC × SO in Study 2a; *df* = 1, 213 for GC; *df* = 2, 213 for SO and GC × SO in Study 2b.)

For perceived sexual orientation, we found significant main effects of gender conformity and sexual orientation as well as an interaction effect. The main effect of gender conformity indicated that GNC targets were perceived as more likely to be gay than GC targets (see Tables 1 and 3). The main effect of sexual orientation indicated that participants perceived targets described as gay (*M* = 4.50, *SD* = 1.90) as more likely to be gay than those described as heterosexual (*M* = 3.55, *SD* = 1.81); *p* = .007, *d* = 0.51. Perceptions of gay and unspecified targets (*M* = 3.90, *SD* = 2.02) did not significantly differ, nor did perceptions of heterosexual and unspecified targets differ; *p*s ≥ .199, *d*s ≤ 0.31. This effect was qualified by a significant interaction with gender conformity. Tukey‐corrected post‐hoc contrasts showed that, for GC targets, participants' attributions followed the explicitly provided sexual orientation information, with gay targets perceived as more likely to be gay than heterosexual and unspecified targets, *p*s ≤ .001, *d*s ≥ 0.81. In contrast, for GNC targets, perceived sexual orientation did not differ across conditions, *p*s ≥ .962, indicating that gender non‐conformity attenuated the influence of explicit sexual orientation information. See Supplementary Materials for full statistics.

###### Main analyses

Across all vulnerability‐related measures, 2 (gender conformity) × 3 (sexual orientation) ANOVAs revealed a consistent main effect of gender conformity, with GNC male targets being perceived as more vulnerable, more likely to have a history of victimization, easier targets of victimization and as more likely to be victimized. Neither the main effects of sexual orientation nor the GC × SO interaction effect were significant. On both measures of victim blaming, no significant main effects or interactions were found^4^ (see Table 3).

###### Exploratory analyses

As no effect of our independent variables on victim blaming was found, we explored correlations between vulnerability, victimization susceptibility and victim‐blaming judgements. Zero‐order correlations revealed that perceived vulnerability was positively associated with both direct, *r* = .36, *p* < .001, and indirect victim blaming, *r* = .20, *p* = .002. In contrast, victimization susceptibility showed weaker and less consistent associations with victim blaming, with only victimization ease being moderately related to direct victim blaming, *r* = .15, *p* = .020 (see Supplementary Materials for full correlations).

### Study 2b

#### Method

##### Sample

Participants completed the survey via Clickworker. After excluding 46 participants who failed the attention check, the final sample consisted of 219 participants (*M*_age_ = 37.55 years, *SD*_age_ = 11.04 years; 106 women, 110 men, 3 non‐binary; 197 heterosexual, 7 gay/lesbian, 14 bisexual, 1 ‘other’; 66.67% with university degree; 10% were students). This was below our target sample (*N* = 265) based on $\eta_{p}^{2}$ = .04 effect size for the gender conformity and sexual orientation interaction. A sensitivity power analysis (G*Power; *α* = .05, 1 − *β* = .80, total sample size = 219, numerator *df* = 2, number of groups = 6; Faul et al., 2007) indicated we could detect an effect of $\eta_{p}^{2}$ = .045 for the study's main analysis.

##### Procedure and materials

The procedure and materials were identical to those in Study 2a, except that gender conformity was manipulated via audio recordings using the same stimuli as in Study 1b. Scales had good reliability: perceived vulnerability (*α* = .78), direct and indirect victim blaming (*α*_DVB_ = .91; *α*_IVB_ = .92).

#### Results

We conducted a 2 (gender conformity: GC vs. GNC) × 3 (sexual orientation: gay vs. heterosexual vs. unspecified) between‐subjects ANOVAs on our variables.

##### Manipulation checks: Gender conformity and perceived sexual orientation

On gender conformity, a significant main effect of gender conformity indicated that GNC speakers were perceived as less masculine than GC speakers. The main effect of sexual orientation and the interaction effect were not significant (see Tables 1 and 3).

For perceived sexual orientation, significant main effects of gender conformity and sexual orientation emerged, while the interaction effect was not significant (see Table 3). The main effect of sexual orientation indicated that targets described as gay (*M* = 3.86, SD = 1.92) were perceived as more likely to be gay than those described as heterosexual (*M* = 2.96, *SD* = 1.93), *p* = .017. Perceptions of unspecified targets (*M* = 3.53, *SD* = 2.06) did not significantly differ from heterosexual targets nor from gay targets, *p* ≥ .186. In addition, a main effect of gender conformity indicated that GNC targets were perceived as more likely to be gay than GC targets (see Table 1). See Supplementary Materials for full statistics.

##### Main analyses

GNC male speakers were perceived as more vulnerable, more likely to have a history of victimization, easier targets of victimization and more likely to be victimized than GC male speakers. Neither the main effect of sexual orientation nor the interaction effect was significant (see Table 3).

On indirect victim blaming, we found that participants imagined others to blame more GNC (*M* = 3.04, *SD* = 1.21) than GC targets (*M* = 2.65, *SD* = 1.22). However, no such effect emerged on direct victim blaming.^5^ Main effects of sexual orientation and interaction effects were also not significant (see Table 3).

##### Exploratory analyses

Replicating the pattern from Study 2a, zero‐order correlations revealed that perceived vulnerability was positively associated with both direct, *r* = .25, *p* < .001, and indirect victim blaming, *r* = .25, *p* < .001, while victimization susceptibility was weakly and less consistently related to direct victim blaming, *p*s ≥ .075, and showed moderate associations to indirect victim blaming, *r*s ≤ .21, *p*s ≤ .003 (see Supplementary Materials for full correlations).

### Discussion

In line with Study 1, GNC male targets were consistently perceived as more vulnerable and more susceptible to victimization than GC targets across both Studies 2a and 2b. We found no significant effects of explicit sexual orientation or interactions with gender conformity. This pattern was consistent across both studies, suggesting that the influence of gender conformity on vulnerability perceptions is robust across modalities and in the presence of explicit sexual orientation information.

In Study 2a, explicit sexual orientation influenced perceived sexual orientation among GC but not GNC targets, suggesting that participants did not fully rely on the explicit information when evaluating GNC targets. This may partly account for the limited effects of explicit sexual orientation on vulnerability‐related perceptions, although the interaction on perceived sexual orientation was not replicated in Study 2b.

Overall, we found no significant effects of gender conformity on direct victim blaming. For indirect victim blaming, however, GNC targets were blamed more than GC targets in Study 2b, whereas no such effect emerged in Study 2a. We found no significant effects of sexual orientation or its interaction with gender conformity on either measure. However, zero‐order correlations revealed that perceived vulnerability was positively associated with both direct and indirect victim blaming. These findings do not support the notion that vulnerability is related to less blame, as predicted by moral typecasting models (i.e. Gray & Wegner, 2009). Rather, they are consistent with blame theories suggesting that vulnerability is associated with inferences about individuals' capacity to foresee and prevent harm and, thus, increased blame (see Malle et al., 2014; Niemi & Young, 2014).

## GENERAL DISCUSSION

This research examined whether behavioural cues of gender conformity and explicit sexual orientation information influenced perceptions of vulnerability, victimization susceptibility and victim blaming. Across four studies, we found that gender non‐conforming (vs conforming) male targets were perceived as more vulnerable and susceptible to victimization, confirming Hypothesis 1. In Study 1, gender non‐conformity (vs conformity) also elicited inferences that male targets were gay, activating intersecting social categories, which were associated with greater vulnerability‐related perceptions. In Study 2, we found no significant interaction effect of explicit sexual orientation information on the effects of gender (non‐)conformity, not supporting Hypothesis 2. Evidence for Hypothesis 3 was less consistent: gender conformity did not significantly affect direct victim blaming, whereas GNC targets received higher indirect blame than GC targets when presented as victims of harassment in Study 2b but not Study 2a.

This research provides useful insights into the factors influencing social perceptions related to victimization when intersecting categories, inferred or explicit, are at play. First, we extend work on stereotyping of men based on their gender conformity and sexual orientation, which has primarily examined dimensions such as warmth and competence (see Fasoli & Hegarty, 2020; Klysing, 2023; McCarty & Burt, 2024; Weißflog & Grigoryan, 2024), to perceptions of vulnerability. Moreover, we focused on gender conformity conveyed by non‐verbal cues (e.g. appearance, gestures and voice) rather than relying on verbal descriptions of the target as gender non‐conforming (e.g. Rodrigues, 2024). Thus, we complement research on vulnerability (e.g. Blaskovits & Bennell, 2019; Book et al., 2013; Fulham et al., 2020; Wheeler et al., 2009), which has typically considered non‐verbal cues associated with vulnerability without considering cues signalling gender or other social categories.

Second, we examined gender conformity and sexual orientation from two complementary perspectives. Although both dimensions have been linked to victimization and victim blaming (e.g. Bränström et al., 2022; Capezza & Arriaga, 2008; Davies et al., 2006, 2008, 2009; Davies & Hudson, 2011; Mason et al., 2025; Plumm et al., 2010), they are strongly intertwined in stereotyping (Miller, 2018) and their respective contributions to person perception and judgements have rarely been disentangled. We first examined sexual‐orientation inferences from behavioural cues of gender conformity, consistent with gender inversion assumptions (Kite & Deaux, 1987) and then orthogonally manipulated gender conformity and explicitly stated sexual orientation. Across studies, gender conformity consistently shaped impressions of vulnerability and victimization susceptibility, whereas we found no significant effects of sexual orientation on these outcomes or interactions between the two.

However, gender conformity did not directly influence blame judgements. This pattern suggests that impression formation and blame rely on different informational requirements. Stereotype activation based on minimal cues appears to operate quickly and automatically, allowing observers to infer characteristics such as vulnerability from limited information. In contrast, blame involves a more evaluative process that requires additional information about the target's capacity and circumstances of the event (Malle et al., 2014; Niemi & Young, 2014). In the absence of clear information, participants may have been reluctant to directly assign blame. Another explanation may lie in the nature of the victimization context examined. Prior work has largely focused on sexual violence, accidents or disease‐related scenarios (e.g. Correia et al., 2001; Pinciotti & Orcutt, 2021; Twardawski et al., 2025), which often provide clearer information about harm, intent and responsibility. Our verbal harassment scenario was moderately severe (e.g. without physical or sexual aggression) and somewhat ambiguous (i.e. unclear perpetrator's intent), which may have prevented participants' willingness to assign blame. Given that victim blaming depends on a complex interplay of situational‐, victim‐, perpetrator‐ and observer‐related factors (see Gravelin et al., 2019; Van der Bruggen & Grubb, 2014; Witte & Flechsenhar, 2025), this context may have provided conditions under which blame was not readily assigned based on minimal or explicit cues alone.

Despite this, perceived vulnerability was positively associated with victim blaming in Studies 2a and 2b, suggesting that higher vulnerability ascriptions are linked to stronger blame‐related judgements. This pattern is consistent with contemporary blame theory insofar as vulnerability may inform assumptions about victims' capacity and obligation to foresee and prevent harm (Malle et al., 2014; Niemi & Young, 2014). It contrasts with moral typecasting, according to which vulnerable targets should be perceived as ‘moral patients’ and consequently blamed less (Gray et al., 2012; Gray & Wegner, 2009). Although moral cognition is strongly organized around perceptions of harm and victimhood (Gray & Kubin, 2024), vulnerability in victimization contexts may not primarily signal deservingness of protection but may instead increase expectations that individuals should anticipate and avoid harm, thereby shifting judgements from protection to responsibility.

Finally, our findings contribute to understanding intersectionality in victimization contexts. They provide evidence that perceptual cues are integrated to infer intersecting social categories and activate stereotypes (see Freeman & Ambady, 2011) while the findings are consistent with the possibility that different sources of information are weighted differently when forming impressions. Although gender non‐conformity prompted assumptions that the targets were gay, gender conformity consistently shaped vulnerability‐related inferences when sexual orientation was explicitly provided. This pattern can be interpreted through the intersectional lens approach (Petsko et al., 2022; Petsko & Bodenhausen, 2019), according to which category prioritization depends on salience, accessibility and contextual fit. This aligns with work by Petsko and Vogler (2024), showing that gender norm violations can be more diagnostic than other social category cues (i.e. the target's ethnicity) when evaluating male targets. Although category prioritization and lens activation were not directly assessed, the observed pattern is consistent with these processes. One explanation for this pattern is that gender is a highly accessible social category (Klysing, 2023; Weißflog & Grigoryan, 2024; Zarate & Smith, 1990; Zhang et al., 2018), and masculinity, in particular, is perceived as precarious and must be continually demonstrated (see Bosson et al., 2009). As a result, deviations from gender norms may be especially salient and diagnostic when forming impressions of men. Consistent with intersectionality research on prototypicality (e.g. Carnaghi et al., 2021; Klysing et al., 2021; Petsko & Bodenhausen, 2019; Purdie‐Vaughns & Eibach, 2008), particularly given that masculinity defines the prototypical male category, gender non‐conformity may therefore have been more *accessible* and diagnostic than other social category information, even when they lead to assumptions of a gay identity. Moreover, gender non‐conformity, more than sexual orientation per se, may have provided a better *fit* – namely, been more contextually relevant – when forming vulnerability‐related impressions, as vulnerability and victimization are associated with a lack of strength and agency, which contradicts stereotypical expectations of men (Eagly et al., 2019; Kincaid, 2022) and, thus, the male prototype.

### Limitations and future research

Several limitations warrant consideration. The studies focused exclusively on male targets. Given evidence that gender conformity shapes victim blaming towards women (e.g. Capezza & Arriaga, 2008), and sexual orientation matters in judgements towards victims of sexual violence (e.g. Dyar et al., 2019; Wakelin & Long, 2003), future work should extend the paradigm to additional gender groups, including women and other gender minorities. Our research also limited the targets to young White men. To expand on the intersectionality perspective, future research should consider additional social identities and markers (e.g. ethnicity, age and social status) that can be conveyed explicitly or via perceptual cues (e.g. face and accent). This would help establish whether the more consistent effects of gender conformity observed here extend beyond categories closely associated with gender expression (e.g. sexual orientation) to those less directly associated with it (e.g. age and social class), and which intersectional lens becomes relevant.

Although the studies were powered to detect the pre‐registered interaction effects, the available sample sizes provided limited sensitivity to smaller effect sizes, particularly in Study 2b, which fell below the pre‐registered target sample. Thus, the absence of significant main effects of sexual orientation and interactions with gender conformity does not rule out smaller effects. Future work should replicate the studies with bigger samples.

Moreover, sexual orientation was manipulated through explicit description rather than self‐disclosure by the target. Although this approach allowed us to contrast explicit and inferred cues, a more immersive manipulation – such as narrative self‐identification or relational context (see an example by De keersmaecker & Roets, 2020) – may produce stronger identity activation. Additionally, presenting both explicit and implicit cues in equivalent modalities (e.g. both via video or both via audio) would allow for a clearer assessment of their relative influence.

Also, our verbal harassment scenario was intentionally ambiguous. Intersectional lens approaches suggest that context determines which category dimension becomes most diagnostic in forming impressions and drawing conclusions from the given context. In forms of victimization that afford certain attitude or personality traits predicting blame judgements (e.g. sexual violence), or in contexts where specific social cues are normatively salient (e.g. a gay bar; see Plumm et al., 2010), specific cues may exert different effects. Future research should therefore systematically vary contextual affordances to determine when different interpretative lenses become relevant to vulnerability inferences and blame judgements.

Finally, we assessed direct and indirect victim blaming using explicit measures, making individuals reluctant to express such judgements, especially in ambiguous situations. Future work should incorporate behavioural or indirect measures to better capture how vulnerability perceptions translate into responses beyond self‐reported judgements (e.g. cognitive‐effort measures, see Sassenrath et al., 2023).

## CONCLUSION

This research shows that minimal behavioural cues of gender conformity shape perceptions of vulnerability and victimization even when sexual orientation information is available. Across studies, gender non‐conforming men were consistently perceived as more vulnerable and susceptible to victimization. Notably, we found neither significant effects of explicit sexual orientation nor interactions with gender conformity on vulnerability‐related perceptions, suggesting that gender prototypicality may play a more prominent role than stated identity information in impression formation concerning victimization.

Moreover, vulnerability was positively associated with victim blaming. Perceived vulnerability was associated with assumptions about victims' capacity and obligation to foresee and prevent harm. Thus, vulnerability ascriptions can have negative downstream consequences for blaming victims. Understanding how vulnerability is inferred from minimal cues, and how such perceptions correspond to evaluations of victims, is critical for addressing biased judgements and for developing more nuanced accounts of when and why victim blaming occurs.

## AUTHOR CONTRIBUTIONS

**Philipp Agostini:** Conceptualization; methodology; investigation; data curation; formal analysis; project administration; writing – original draft; visualization; writing – review and editing. **Morgana Lizzio‐Wilson:** Conceptualization; writing – review and editing. **Mario Gollwitzer:** Writing – review and editing. **Fabio Fasoli:** Conceptualization; methodology; investigation; writing – original draft; project administration; writing – review and editing; supervision.

## FUNDING INFORMATION

The authors received no financial support for the research, authorship and/or publication of this article.

## CONFLICT OF INTEREST STATEMENT

The authors declare no potential or actual conflicts of interest with respect to the research, authorship and/or publication of this article.

## ETHICS STATEMENT

The studies were approved by the University Ethics Committee (UEC) of the University of Surrey (EGA ref.: 0116). Informed consent was obtained from all participants.

## AI STATEMENT

During the preparation of this work, the authors used ChatGPT (OpenAI; GPT‐5.3 and GPT‐5.6) in order to support data analysis, statistical coding and to improve readability and flow of the manuscript. After using this tool, the authors reviewed and verified all outputs as needed and take full responsibility for the content of the publication.

## Data Availability

The pre‐registration and analysis plan is available under https://osf.io/buzvq for Studies 1a and 1b and under https://osf.io/qzu6g for Studies 2a and 2b. All measures, analysis scripts, code and outputs, as well as Supplementary Materials, are available online at https://osf.io/c4qfm for all studies.

## Footnotes

^1^
We note that alternative theoretical perspectives could lead to different predictions regarding the effect of gender conformity on victim blaming. Hypothesis 3 was primarily informed by the contemporary blame theory and prior empirical findings, including our own preliminary (unpublished) data.

^2^
For all studies, we deviated from the preregistered analyses and report results including participants who did not identify as heterosexual. Analyses only on heterosexual participants showed similar patterns of results (see Supplementary Materials for a detailed overview).

^3^
A sensitivity power analysis conducted in G*Power (*α* = .05, 1 − *β* = .80, sample size group 1 = 71, sample size group 2 = 73; Faul et al., 2007) suggested we could detect an effect of Cohen's *d* = 0.416 for our main analysis with this sample.

^4^
When conducting the preregistered analyses (*t*‐tests), the effects for both victim‐blaming measures were non‐significant, *t*s ≤ + − 0.09, *p*s ≥ .178, *d*s ≤ + − 0.12.

^5^
When conducting the preregistered analyses (*t*‐tests), we found that GNC targets were blamed more than GC targets in both direct, *t*(208.82) = −1.69, *p* = .046 (one‐sided), *d* = 0.23, and indirect victim blaming, *t*(216.72) = −2.34, *p* = .010 (one‐sided), *d* = 0.32.
